# Quercetin Suppresses Drug-Resistant Spheres via the p38 MAPK–Hsp27 Apoptotic Pathway in Oral Cancer Cells

**DOI:** 10.1371/journal.pone.0049275

**Published:** 2012-11-12

**Authors:** Su-Feng Chen, Shin Nieh, Shu-Wen Jao, Chia-Lin Liu, Chien-Hua Wu, Yun-Ching Chang, Chin-Yuh Yang, Yaoh-Shiang Lin

**Affiliations:** 1 Department of Dental Hygiene, China Medical University, Taichung, Taiwan; 2 Department of Pathology, Tri-Service General Hospital & National Defense Medical Center, Taipei, Taiwan; 3 Division of Colon and Rectal Surgery, Tri-Service General Hospital & National Defense Medical Center, Taipei, Taiwan; 4 Graduate Institute of Life Sciences, National Defense Medical Center, Taipei, Taiwan; 5 Department of Dentistry, Shuang Ho Hospital & Taipei Medical University, Taipei, Taiwan; 6 Department of Otolaryngology-Head and Neck Surgery, Tri-Service General Hospital & National Defense Medical Center, Taipei, Taiwan; 7 Department of Otolaryngology-Head and Neck Surgery, Chung Shan Medical University, Taichung, Taiwan; University of South Florida College of Medicine, United States of America

## Abstract

**Background:**

Treatment failure in oral squamous cell carcinoma (OSCC) leading to local recurrence(s) and metastases is mainly due to drug resistance. Cancer stem cells (CSCs) are thought be responsible for the development of drug resistance. However, the correlations between CSCs, drug resistance, and new strategy against drug resistance in OSCC remain elusive.

**Methods:**

A drug-resistant sphere (DRSP) model was generated by using a nonadhesive culture system to induce drug-resistant cells from SCC25 oral cancer cells. A comparative analysis was performed between the parent control cells and DRSPs with a related treatment strategy focusing on the expression of epithelial–mesenchymal transition (EMT)-associated markers, drug-resistance-related genes, and CSC properties *in vitro*, as well as tumorigenicity and the regimen for tumor regression *in vivo*.

**Results:**

Our data show the presence of a phenomenon of EMT with gradual cellular transition from an epithelioid to mesenchymal-like spheroid morphology during induction of drug resistance. The characterization of DRSPs revealed the upregulation of the drug-resistance-related genes *ABCG2* and *MDR-1* and of CSC-representative markers, suggesting that DRSPs have greater resistance to cisplatin (Cis) and stronger CSC properties compared with the control. Moreover, overexpression of phosphorylated heat-shock protein 27 (p-Hsp27) via the activation of p38 MAPK signaling was observed in DRSPs. Knockdown of Hsp27 decreased Cis resistance and induced apoptosis in DRSPs. Furthermore, an inhibitor of Hsp27, quercetin (Qu), suppressed p-Hsp27 expression, with alterations of the EMT signature, leading to the promotion of apoptosis in DRSPs. A xenographic study also confirmed the increase of tumorigenicity in DRSPs. The combination of Qu and Cis can reduce tumor growth and decrease drug resistance in OSCC.

**Conclusions:**

The p38 MAPK–Hsp27 axis plays an important role in CSCs-mediated drug resistance in OSCC. Targeting this axis using Qu combined with Cis may be a treatment strategy to improve prognosis in patients with OSCC.

## Introduction

Oral squamous cell carcinoma (OSCC) is one of the most common and lethal head and neck malignancies in Taiwan and worldwide [1], [2]. Current treatments for oral cancer are of limited efficacy in preventing tumor recurrence and progression, and a significant proportion of patients develop local invasion and metastases [3]. The prognosis of patients with oral cancer is relatively poor despite recent therapeutic advances [4]. Cisplatin (Cis)-based chemotherapy is the main treatment for patients with advanced oral cancer and is used at least for palliative purposes. However, the efficacy of chemotherapy is limited because of *de novo* drug resistance. Therefore, it is important to elucidate the mechanisms underlying the mediation of chemoresistance and to develop a new strategy for the treatment of OSCC.

The concept of cancer stem cells (CSCs) was proposed decades ago based on the similarities between cancer cells and normal stem cells [5]. The existence of CSCs was first characterized in the context of leukemia [6]. The CSC hypothesis suggests that tumors comprise a small population of cells that possess tumor-forming and self-renewing abilities [7]. Accumulating evidence demonstrated that CSCs not only can lead to cancer relapse and metastasis but they also contribute to the resistance of tumors to chemotherapy [8].

ABCG2 is the most well-known gene and is expressed in a wide variety of stem cells which has been served as a marker for stem cells from various sources [9], [10]. MDR1 expression has been also reported in various types of chemoresistant tumor phenotypes [11], [12]. Multiple mechanisms have been proposed for cisplatin resistance recently [13], [14] based on the fact that cisplatin acts a multiple cellular targets representing diverse signal transduction pathways [13]. One of the most important mechanisms other than regulation of *ABCG2* and *MDR-1* in association with resistance to cisplatin is reduced intracellular accumulation due to impaired drug intake involving in maintenance of copper homeostasis such as Human copper transporter 1 and the two copper efflux transporters ATP7A and ATP7B which regulate the efflux of cisplatin [15]–[17].

Despite the link between the induction of CSCs in tumor cells and the acquisition of drug resistance and recurrence of tumors, the mechanism underlying these phenomena remains largely unknown.

Heat-shock proteins (Hsps) are generally induced by environmental stress and function as molecular chaperones, which are responsible for maintaining the correct conformation of other proteins. Hsps are classified into high-molecular-weight Hsps, such as Hsp90 and Hsp70, and low-molecular-weight Hsps, including Hsp27 [18]. In addition to its conventional function as a chaperone, Hsp27 has been reported to be overexpressed in breast, ovarian, and head and neck cancers [19]–[21]. The expression of Hsp27 has been associated with poor prognoses and survival rates in patients with different cancers. Moreover, Hsp27 has been demonstrated to be associated with chemoresistance and induction of cancer cells bearing stem-cell-like properties in breast cancer and many other malignancies. However, reports of the involvement of Hsp27 in drug resistance in and prognosis of, OSCC are limited. Quercetin (Qu) is the principal flavonoid compound (3,30,40,5,7-penta-hydroxy-flavanone) commonly extracted from cranberries, blueberries, apples, and onions. It possesses a wide spectrum of bio-pharmacological properties [22] and may offer promising new options for the development of more effective chemopreventive and chemotherapeutic strategies because of its powerful antioxidant and free-radical-scavenging properties [23]. A previous report also revealed that Qu plays a role as an inhibitor of Hsp synthesis and has protective effects in mouse liver injury for cellular homeostasis [24]. However, the details of the relationship between Qu and Hsp27 regarding their involvement in drug resistance in cancer need further investigation.

Our group previously established a novel nonadhesive sphere culture system that allowed us to purify and enrich a population of OSCC cells with stem-cell-like properties [25]. Here, we took advantage of this well-established culture system to investigate the resistance of oral cancer to Cis and its underlying molecular mechanism. Based on the establishment of drug-resistant spheres (DRSPs), the objective of the current study was to validate the possible role of Hsp27 and its associated signaling pathway in the modulation of apoptosis. Furthermore, we chose the combination of Qu and Cis as a potential therapeutic agent to explore how Qu interacts with Hsp27 and the mechanism underlying the effective Qu-mediated suppression of chemoresistance and tumor growth in OSCC. This study may provide an insight into drug resistance and new treatment strategies against drug resistance, which could be used to improve prognosis and survival in patients with OSCC.

## Methods

### Cell and Sphere Culture

The human tongue cancer cell line SCC25, was obtained from the ATCC (ATCC number: CRL-1628) cultured in RPMI supplemented with 10% fetal bovine serum (FBS) at 37°C in the presence of 5% CO_2_ . The cell was cultured in culture plastic wares with nonadhesive surface. 10 cm dish are made of nonadhesive for cells by coating with agarose thin films. Cells were plated at a density of 5×10^4^ live cells/10 cm dish, and the culture medium was changed every other day until the sphere formation, as seen in our previous reported [25].

### Induction of Drug Resistance Cells

SCC25 parental cells were plated at a density of 5×10^4^ live cells/10 cm dish, continued treat with Cis were added at the final concentrations (1, 5, 10, 20, and 30 µM) for three month consequently. After treatment cells were harvested and maintaining low concentration Cis (0.5 µM) culture medium and the culture medium was changed every other day.

### Cell Viability Analysis

The Cis was added at a dose rate of (5, 10, 20, and 30 µM). Cells were seeded on 6-well plates at a density of 2×10^4^ per well in medium, after treatment with Cis for 48 h, and analyzed by the MTT assay (Sigma-Aldrich).

### RNA Interference

The siRNA oligos of Hsp27 consisted of three targets specific siRNAs designed to knockdown gene expression. The sequence of three targets were showed in below: sc-29350A: sense:5'- GAGUGGUCGCAGUGGUUAGtt -3'; antisense: 5'-CUAACCACUGCGACCACUCtt-3'); sc-29350B: sense: GACGAGCUGACGGUCAAGAtt; antisense: UCUUGACCGUCAGCUCGUCtt; sc-29350C: Sense: CCACGCAGUCCAACGAGAUtt; Antisense: AUCUCGUUGGACUGCGUGGtt or negative control siRNA oligos was purchased from Santa Cruz Biotechnologies, Inc. 2×10^6^ cells were transfected with a final concentration (100 nM) of Hsp27 siRNA using Lipofectamine 2000 for 48 h to detect protein level. Cells which were transfected with non-specific siRNA (MOCK group) were parallelism demonstrated.

### Western Blot Analysis

Whole cell lysates were separated by electrophoresis on 12% SDS–PAGE and transferred to polyvinylidene fluoride membrane. The membranes were blocked with 5% nonfat milk at room temperature for 1 h. The primary antibodies were used: Hsp27 (Santa Cruz; sc-1049; 1∶1000) GAPDH (ab9482; 1∶5000 dilution) (Abcam, Cambridge, MA, USA), Oct-3/4 (sc-8630; 1∶1000), NANOG (sc-81961; 1∶1000), SOX2 (sc-17320; 1∶500) (Santa Cruz Biotechnology), ATP-binding cassette sub-family G member 2 (ABCG2) (sc-8630; 1∶1000), MDR-1 (sc-8630; 1∶1000), p38MAPK (sc-8630; 1∶1000), pp38MAPK(sc-8630; 1∶1000),capase-3 (sc-8630; 1∶1000) and PARP (sc-81961; 1∶1000) in TBST buffer containing 3% nonfat milk at 4°C overnight and subsequently with anti-mouse and rabbit anti-goat secondary antibody conjugated with peroxidase (1∶1000) (Santa Cruz Biotechnology) at 25°C for 1 h. The immunoblots were developed using an enhanced chemiluminescence system, and the luminescence was visualized on X-ray film.

### *In vivo* Tumorigenic Assay

The *in vivo* tumorigenicity study was performed following local ethics committee guidelines that had full accreditation awarded by the Association for Assessment and Accreditation of Laboratory Animal Care in the National Defense Medical Center. Mice were kept at 18–26°C, 30–70% humidity, and independently air- conditioned under a 12 h dark/12 h light cycle for 7 days before xenograft injection. The parental OSCC cells and spheres were injected into the BALB/c nude mice (6 weeks). The cell suspension (100 ml) was injected subcutaneously in each mouse with different cell numbers from 1×10^6^, 1×10^5^, and 1×10^4^ cells. Tumors were formed in 7 days after injection. Tumor sizes were monitored and measured weekly according to the formula (length×width^2^)/2. At 30 days after or orthotopic inoculation, mice were euthanized under anesthesia. All of the animals were conformed and approved by the Institutional Animal Care and Use Committee in National Defense Medical Center (IACUC-11-064).

### Immunohistochemistry

Tissue sections or cell block were de-waxed in xylene and rehydrated in alcohol. Antigen retrieval was carried out by incubation in 10 mM citrate buffer (pH 6.0) at 95°C for 40 min. Endogenous peroxidase was blocked with 0.3% hydrogen peroxide for 10 min then incubated with 5% normal horse serum in phosphate-buffered saline (PBS) for 60 min at room temperature to block non-specific antibody reaction. After a wash with Tris-buffered saline plus 0.1% Tween 20(TBST), slides were incubated overnight at 4°C with primary antibodies, E-cadherin (sc-8426; 1∶800) and fibronectin (sc-18825; 1∶500) (Santa Cruz Biotechnology, Inc., CA. USA). After being rinsed in TBST, slides were incubated for 30 min at room temperature with biotinylated secondary antibody followed by streptavidin–biotinylated–enzyme complex (streptABComplexes kit; Dako, Glostrup, Denmark). Subsequently, they were stained with 0.003% 3, 3-diaminobenzidine tetrahydrochloride, counterstained with Mayer’s hematoxylin, dehydrated, and mounted.

### Statistical Analysis

The independent Student's t test or ANOVA was used to compare the continuous variables between groups, whereas the Χ^2^ test was applied for the comparison of dichotomous variable. The level of statistical significance was set at 0.05 for all tests. All statistical analyses were performed using SPSS version 12.0 (SPSS, Inc., Chicago, IL, USA).

## Results

### Characterization of DRSPs in OSCC Cells

**Figure 1:**
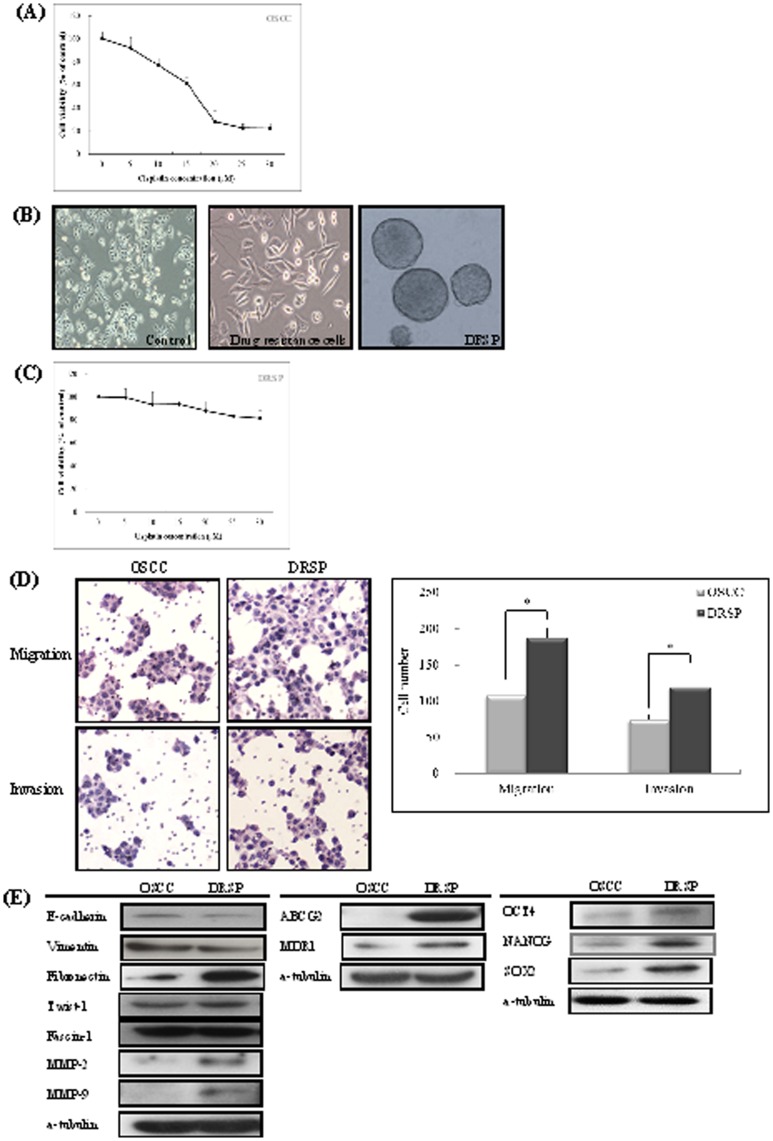
Establishment and characterization of DRSPs and comparison with control cells. (A) Drug-resistant OSCC cells (DRCs) were established via multistep Cis treatment at various concentrations. Only a small proportion of DRCs were maintained in the long term after Cis treatment. (B) DRCs were further cultured using a nonadhesive culture system, which yielded a spheroid phenotype termed drug-resistant spheres (DRSPs). Gradual morphological alterations of DRCs that were indicative of the EMT phenomenon were observed during the induction. (C) After treatment with Cis, DRSP cells were more resistant to Cis than were control OSCC cells. (D) DRSPs were further characterized by increased migration and invasion abilities compared with control cells. (E) Comparisons of EMT- and invasion-associated markers, drug-resistance-related gene products, and CSC-representative markers revealed alterations of the expression of EMT-associated markers and significant upregulation of MMP-2 and MMP-9, drug-resistance-related gene products, and CSC-representative markers in DRSPs compared with control cells.

Drug-resistant cells (DRCs), induced from the SCC25 cell line were first established via multistep Cis treatments at various concentrations (0, 5, 10, 20, and 30 µM). Only a small proportion of DRCs (20%) were maintained in the long term after Cis treatment at 25 µM (Figure 1A). DRCs were further cultured using a nonadhesive culture system, as described in our previous report. Culture for 7 days yielded a spheroid phenotype termed a drug-resistant sphere (DRSP). A gradual morphological alteration of DRCs from an epithelioid, polygonal appearance to a mesenchymal-like, spindle configuration, which is representative of the EMT phenomenon, was observed during the induction from the parent OSCC cells to DRCs (Figure 1B). After treatment with 25 µM Cis for 48 h, DRSPs were much more resistant to Cis than were the control OSCC cells (Figure 1C). In addition to the morphological alterations suggestive of the EMT phenomenon during the induction, DRSPs were characterized by increased migration and invasion abilities compared with control cells (Figure 1D). The characterization of DRSPs using Western blot analysis showed the upregulation of the drug-resistance-related genes *ABCG2* and *MDR-1* and of CSC-representative markers, including OCT4, NANOG, and Sox2, suggesting that DRSPs have greater resistance to Cis and stronger CSC properties compared with control cells. A comparative analysis revealed alterations of EMT-associated markers, including decreased expression of E-cadherin, increased expression of fibronectin and Twist-1, and significantly increased expression of MMP-2 and MMP-9 in DRSPs compared with control cells (Figure 1E).

### Involvement of the p38 MAPK–Hsp27 Antiapoptotic Pathway in Drug Resistance in OSCC

**Figure 2:**
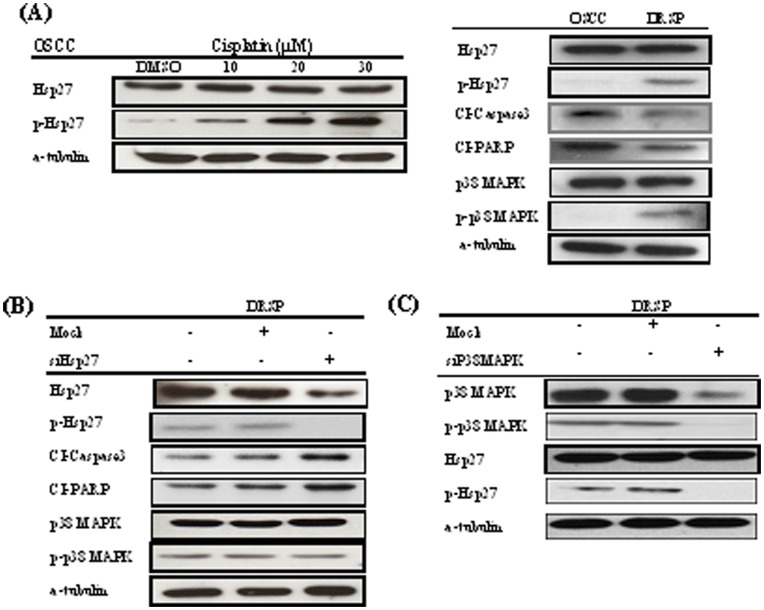
Involvement of the p38 MAPK–Hsp27 antiapoptotic pathway in drug resistance. (A) The expression of p-Hsp27 was dose-dependently correlated with Cis treatment in OSCC cells. P-Hsp27 was correspondently overexpressed via the activation of upstream p38 MAPK signaling in DRSPs. (B) Knockdown of Hsp27 reversely induced the upregulation of CI-caspase 3 and CI-PARP, resulting in increased apoptosis in DRSPs. However, there was no alteration of upstream p38 MAPK. (C) Knockdown of p38 MAPK directly downregulated p-Hsp27, indicating the presence of the p38 MAPK–Hsp27 antiapoptotic pathway in DRSPs.

The expression of p-Hsp27 was dose-dependently correlated with Cis treatment in OSCC cells (Figure 2A). P-Hsp27 was overexpressed via activation of upstream p38 MAPK signaling in DRSPs. An additional study via siRNA of Hsp27 reversely induced the upregulation of CI-caspase 3 and CI-PARP, resulting in the promotion of apoptosis in DRSPs. However, there was no effect on p38 MAPK expression (Figure 2B). Evidence from Western blot analysis showed that the knockdown of p38 MAPK directly downregulated p-Hsp27, indicating the presence of the p38 MAPK–Hsp27 antiapoptotic pathway in DRSPs (Figure 2C).

### Qu-mediated Inhibitory Effect on p-Hsp27 Resulting in Enhancement of Apoptotic Activity in DRSPs

**Figure 3:**
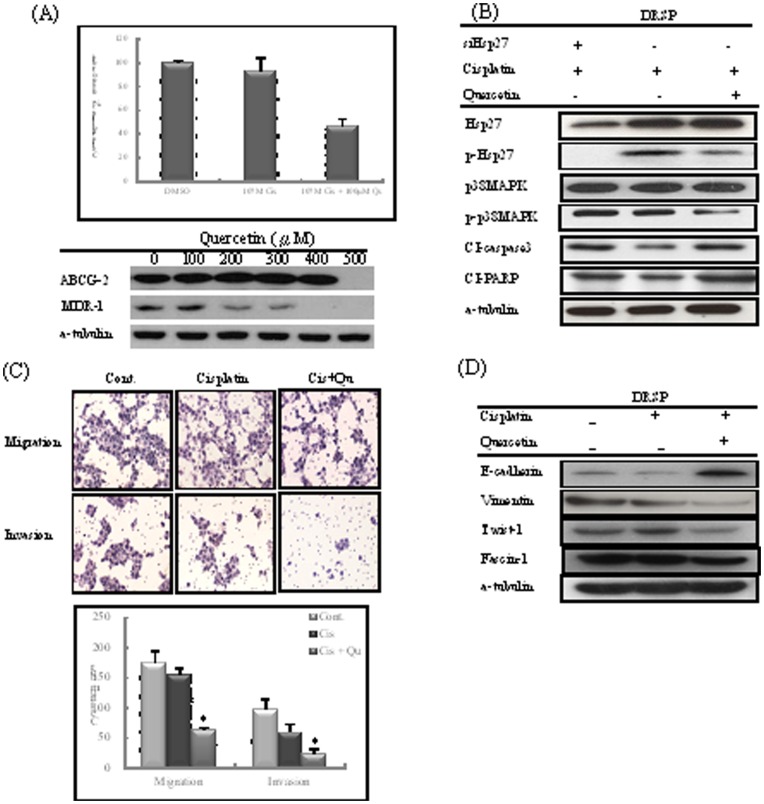
Inhibitory effect of p-Hsp27 and enhancement of apoptosis by Qu in DRSPs. (A) An MTT assay demonstrated that Qu treatment at a dose of 100 µM combined with Cis can suppress DRSPs. Qu attenuated the expression of the products of the drug-resistance-related genes *ABCG2* and *MDR-1* in a dose-dependent manner. (B) Both the knockdown of Hsp27 and the use of Qu combined with Cis effectively induced similar apoptotic effects that led to the decrease of pHsp27 and upregulation of CI-caspase 3 and CI-PARP in DRSPs. (C) Treatment with Qu combined with Cis markedly decreased the migration and invasion abilities in DRSPs. (D) Treatment with Qu combined with Cis reversed the expression of EMT-associated markers and induced the EMT phenomenon.

An MTT assay demonstrated that treatment with 100 µM Qu combined with 10 µM Cis reduced DRSP growth and significantly chemosensitized DRSPs to Cis, as evidenced by a decrease in cell survival after Cis treatment in DRSPs (*P*<0.01). Qu inhibited the products of the drug-resistance genes *ABCG2* at a dose of 500 µM and also gradually attenuated *MDR-1* in a dose-dependent manner. *MDR-1* was more vulnerable to Qu treatment than was *ABCG2* (Figure 3A). The knockdown of Hsp27 or the use of Qu combined with Cis yielded similar apoptotic effects, leading to downregulation of p-Hsp27 and upregulation of CI-caspase 3 and CI-PARP, but had no effect on p38 MAPK in DRSPs (Figure 3B). Migration and invasion assays showed that treatment with Qu combined with Cis markedly decreased migration and invasion abilities in DRSPs (Figure 3C). Treatment with Qu combined with Cis reversed the expression of EMT-associated markers, yielding upregulation of E-cadherin and downregulation of vimentin, Twist-1, and fascin-1 (Figure 3D).

### Inhibition of Tumor Growth and Attenuation of Cis Resistance by Qu *in vivo*

**Figure 4:**
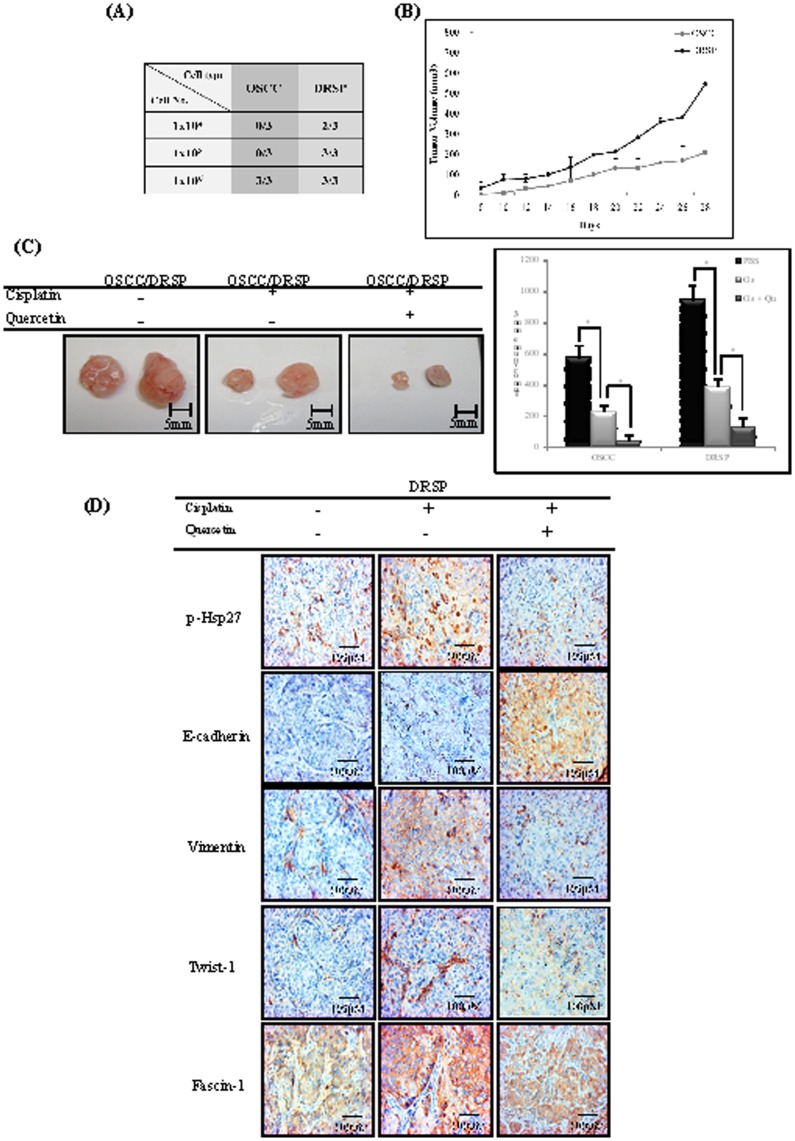
Qu-mediated inhibitory effect of tumor growth and drug resistance *in vivo.* (A) A higher capability of tumorigenicity was shown in DRSPs compared with control cells. (B) The size and volume of DRSP-induced tumors were significantly higher than those of tumors induced by control cells. Growth differences of the tumors generated were observed in a time-dependent manner in DRSPs vs. control cells (*P*<0.05). (C) Gross appearance of a representative tumor formed via inoculation of parental cells and dissociated DRSPs into NOD/SCID mice (n = 3 in each group). Mice treated with Qu combined with Cis disclosed statistically significant growth inhibition compared with the group treated with Cis alone and the control group (*P*<0.05). (D) A comparative analysis of corresponding immunohistochemical results for p-Hsp27 and EMT-associated markers taken from the tumor nodules of the NOD/SCID mice after treatment with Qu combined with Cis revealed a marked downregulation of p-Hsp27, vimentin, Twist-1, and fascin-1 and upregulation of E-cadherin in DRSPs (magnification, 100×).

To confirm the tumor-initiating capabilities of spheres *in vivo*, both parental cells and spheres were injected into male nude mice for an analysis of transplanted tumorigenicity. The results of this experiment showed a higher capability for tumorigenicity in DRSPs compared with control cells. DRSPs gave rise to tumors after injection of 1×10^4^ cells into mice (two out of three mice). In contrast, more control cells were needed to generate tumors (injection of 1×10^6^ cells into mice; three out of three mice), suggesting that DRSPs are enriched for tumor-initiating cells by at least 20-fold compared with control cells (Figure 4A). The size and volume of DRSP-induced tumors were significantly higher than those of tumors induced by control cells. Growth differences in the tumors generated were gradually observed in a time-dependent manner after injection of DRSPs vs control cells (*P*<0.05) (Figure 4B). Mice treated with Qu combined with Cis exhibited significant inhibition of tumor growth compared with the group treated with Cis alone and the control group (Figure 4C). A comparative analysis of corresponding immunohistochemical results for p-Hsp27 and the EMT-associated markers taken from the tumor nodules of the mice after treatment with Qu combined with Cis revealed a marked downregulation of p-Hsp27, vimentin, Twist-1, and fascin-1 and upregulation of E-cadherin in DRSPs compared with the group that did not receive treatment (Figure 4D).

## Discussion

Cis-based chemotherapy is widely used for the clinical treatment of advanced cancer. It improves the prognosis and survival rate in patients with OSCC. The development of Cis resistance has been considered a major cause of tumor recurrence, leading to the failure of clinical treatment. The role of CSCs within tumors in the induction of chemoresistance during chemotherapy is known [26]. Therefore, the identification of the molecular mechanism that mediates chemoresistance seems crucial for the verification of the correlation between recurrence, Cis resistance, and CSCs in OSCC. In this regard, we used a nonadhesive sphere culture system to isolate cells with stem-cell properties among Cis-resistant cells and generated DRSPs. During the induction of DRSPs, we noticed an interesting and special EMT phenomenon, as evidenced by the gradual morphological alteration from the parent OSCC cells to DRCs. This implies that DRCs possess the property of drug resistance via EMT. Previous studies have showed that EMT is the pivotal mechanism of cancer aggressiveness and has shown to correlate with the development of chemoresistance [27]–[29]. These well-formed DRCs with a mesenchymal-like phenotype may undergo further sphere formation in a nonadhesive sphere culture system. The adversity met by spheres in a nonadhesive, suspended condition can not only be stimulated by EMT but also encourage the enrollment of the potential of CSC properties, as described in our previous study and others [25], [30].

Based on our study, in addition to the morphological alterations along with the changes to the expression of EMT-associated markers, DRSPs were characterized by the upregulation of *ABCG2* and *MDR-1*. Those two genes are well-known and are expressed in a wide variety of CSCs which have been served as chemoresistant markers [9]. DRSPs also demonstrated the overexpression of CSC-representative and stemness markers, including OCT4, NANOG, and Sox2, which indicates that DRSPs exhibit greater Cis-resistance ability and stronger CSC properties than do control OSCC cells. These data not only demonstrate that DRSPs are a useful model for a drug resistance study but also provide good evidence in support of the hypothesis that CSCs contribute to drug resistance in tumor cells, as proved by our study and also reported by others previously [25], [31]. Taking advantage of DRSPs as a study model for drug resistance, we also found a significant increase in the expression of p-Hsp27 in DRSPs, which was negatively correlated with the expression of the CI-caspase 3 and CI-PARP proteins, indicating that p-Hsp27 may participate in the drug-induced antiapoptotic effect. As mentioned earlier, Hsp27 contributes to the malignant properties of cancer cells, including increased tumorigenicity, resistance to treatment, and inhibition of apoptosis [32]. Prognosis indicators, such as Hsp27 which is closely correlated with patients’ survival or response to therapy, may serve as novel therapeutic targets based on their chemoresistance property [33]. There is increasing evidence of the role of Hsp27 in drug resistance and CSC formation in a variety of cancers [29], [34]. In addition, the function of Hsp27 is controlled by posttranslational modification, such as phosphorylation; p38 MAPK signaling is responsible for the phosphorylation of Hsp27, which leads to its functional induction [35]. In the current study, upregulation of the phosphorylation of Hsp27 via the activation of p38 MAPK signaling was observed in DRSPs. It is suggested that the activation of Hsp27 is triggered by a canonical p38 MAPK cascade. Moreover, knockdown of Hsp27 in DRSPs decreased Cis resistance and induced apoptosis via the activation of caspase signaling. Taken together, these results show that, among the numerous signaling pathways, p38 MAPK-dependent signaling may be the critical target of Hsp27. Therefore, Hsp27 plays an essential role in Cis-resistant DRSPs, most likely via the antiapoptotic signaling pathway. An additional study enrolling clinical cases related to the poor prognosis with recurrent or metastatic OSCC is ongoing and aims to dissect the distinct role of Hsp27 on drug-resistance in tissue sections by immunohistochemistry.

A recent study showed that the expression of Hsp27 was increased in lung CSCs and that treatment of these cells with a combination of cisplatin/gemcitabine chemotherapy and the plant flavonoid compound Qu inhibited Hsp27 expression and suppressed tumor growth and the expression of stemness genes, including *Oct4*, *Nanog*, and *Sox2* [24]. Another report demonstrated that Qu inhibits spheroid formation, cell survival, and invasion in prostate cancer stem cells [36]. To reconcile the role of Qu as a coadjuvant in OSCC and an inhibitor of Hsp27, we further validated the hypothesis that Qu exerts its apoptotic activity through the suppression of heat shock factor activity and its associated molecular signaling, which is critical for OSCC, as reported in other cancers [34]. The conclusion that Qu functions as an effective chemopreventive agent in OSCC was based on the evidence that Qu enhances the activity of caspase 3 and PARP, directly influencing the downstream apoptotic pathway involving Hsp27/caspase 3/PARP signaling. We demonstrated that Qu may attenuate the expression of the *ABCG2* and *MDR-1* gene-related products in a dose-dependent manner and reverse the expression of EMT-associated proteins. Either knockdown of Hsp27 or the use of Qu combined with Cis exerted similar apoptotic effects, as evidenced by the downregulation of p-Hsp27 and the upregulation of CI-caspase 3 and CI-PARP in DRSPs. In addition, Qu combined with Cis significantly inhibited cell proliferation, migration, and invasion, which were accompanied by alterations in the expression of fascin-1 and EMT-associated proteins in DRSPs. Furthermore, Qu combined with Cis attenuated the tumorigenicity of DRSPs, indicating its powerful inhibitory effect in reducing tumor growth and decreasing drug resistance *in vivo* as a result of the downregulation of p-Hsp27 and alterations in the EMT phenomenon. To our knowledge, the present study is the first to show that the specifically designed DRSPs are proved to be an applicable model which validates the molecular mechanism that underlies Hsp27-mediated drug resistance and the related treatment strategy using Qu as a coadjuvant both *in vitro* and *in vivo*.

In conclusion, our study provides an insight into the mechanism underlying drug resistance in OSCC. The p38 MAPK–Hsp27 axis plays an essential role in CSC-mediated Cis resistance in oral cancer. Targeting this axis by using Qu combined with traditional chemotherapy may represent a treatment strategy to improve prognosis in patients with OSCC.
